# Cost Analysis in Shoulder Arthroplasty Surgery

**DOI:** 10.1155/2012/692869

**Published:** 2012-12-02

**Authors:** Matthew J. Teusink, Nazeem A. Virani, John A. Polikandriotis, Mark A. Frankle

**Affiliations:** ^1^Shoulder and Elbow Services, Florida Orthopaedic Institute, 13020 N. Telecom Parkway, Tampa, FL 33637, USA; ^2^Department of Shoulder and Elbow Research, Foundation for Orthopaedic Research and Education, 13020 N. Telecom Parkway, Tampa, FL 33637, USA

## Abstract

Cost in shoulder surgery has taken on a new focus with passage of the Patient Protection and Affordable Care Act. As part of this law, there is a provision for Accountable Care Organizations (ACOs) and the bundled payment initiative. In this model, one entity would receive a single payment for an episode of care and distribute funds to all other parties involved. Given its reproducible nature, shoulder arthroplasty is ideally situated to become a model for an episode of care. Currently, there is little research into cost in shoulder arthroplasty surgery. The current analyses do not provide surgeons with a method for determining the cost and outcomes of their interventions, which is necessary to the success of bundled payment. Surgeons are ideally positioned to become leaders in ACOs, but in order for them to do so a methodology must be developed where accurate costs and outcomes can be determined for the episode of care.

## 1. Introduction

The increasing cost of the delivery of health care services in the setting of limited resources has generated increased interest in cost analysis of orthopaedic procedures. Increased analysis of health care costs has taken on a new focus with the passage of the Patient Protection and Affordable Care Act on March 31, 2011 [1]. As a provision of this law, Accountable Care Organizations (ACOs) were introduced under the Medicare Shared Savings Programs. In the current system, there are multiple claims from multiple providers which are all individually paid by the Centers for Medicare and Medicaid Services (CMS). As part of the Bundled Payment initiative of the ACO's, one entity would receive a bundled payment and pay all other parties affiliated with the episode of care from this amount. The goal of this model is to provide an incentive to provide health care services more efficiently while maintaining or improving on the quality of care and therefore increasing the value of the services delivered.

The decision process in warranting surgical management of an elective reconstructive orthopaedic condition requires the assessment of (1) the pathologic mechanical problem, (2) the patient's quality of life (disability), and (3) the orthopaedic surgeon's perceived technical ability to produce a desirable outcome. Since this assessment is the initiation into the episode of care, the orthopeadic surgeon can take on a leadership role in the bundled payment initiative. Given its reproducible nature, shoulder arthroplasty is ideally situated to be a model for the bundled payment initiative. This paper will review the previous cost analysis literature in shoulder arthroplasty and propose a new method of prospective data collection so that orthopaedic surgeons may become leaders in ACOs and the bundled payment initiative.

## 2. Background

 Rising health-care costs have become a central issue in the long-term financial health of the United States. The current percentage of the United States gross domestic product spent on health care has risen to 17.9% in 2010 and is projected to increase as high as 34% by 2040 [2, 3]. A large portion of health care expenditures are related to musculoskeletal care as nearly 30% of the population have a musculoskeletal condition requiring medical care. Chronic shoulder pain is the third most common musculoskeletal complaint after back and knee pain [4].

 Economic evaluations can be classified into four basic categories [5].

1. Cost-minimization analysis incorporates all the costs associated with different interventions to determine which intervention is the least costly. This type of analysis requires that the clinical outcomes of the interventions are the same or similar [6].
2. Cost-effectiveness analysis measures health outcomes in physical or natural health units (e.g., life years gained, patients successfully treated). This analysis is useful when outcomes of interventions vary, but can be expressed in natural health units. There is no value placed on the reported health outcomes. This type of analysis is believed to be more objective because subjective factors such as patient preference are not considered. With a common unit of outcome, different interventions can be compared and reported as cost per unit of outcome [7].
3. Cost-utility analysis compares interventions that result in different outcomes which are expressed as standardized health utility measures. These utilities combine morbidity and mortality to produce a composite index, which places higher value on time spent in good health than on time spent with physical or emotional impairment. The most common health utility measure is the quality-adjusted life-year. This analysis is useful when interventions produce different outcomes or longer survival is brought about at the expense of reduced quality of life [6, 7].
4. Cost-benefit analysis values both costs and outcomes in monetary terms. The outcomes are valued based on what the health-care consumer would be willing to pay for the health services in order to achieve a given outcome or the value of returning a person to the work force after the treatment. The goal is to determine whether the value of the outcome exceeds the value of the resources required [6, 7].

## 3. Cost Analysis in Shoulder Surgery

There has been a great deal of interest of cost analysis within elective orthopaedic surgery [8–11]. Unfortunately, there has been a paucity of literature regarding the costs and cost effectiveness of shoulder surgery. Kuye et al. performed a systematic review on economic evaluations in shoulder pathologies and found only 32 studies in the entire literature which met their criteria. There were only eight studies performed for the common shoulder surgical procedures of rotator cuff repair and shoulder arthroplasty. Over half (17/32) of all of the studies in their review were performed within the past 5 years [6]. Vitale et al. first reported on the cost-effectiveness of rotator cuff repair in 2007 by prospectively collecting cost and outcome data (Health Utility Index, EuroQOL) on patients undergoing rotator cuff repair and found it to be a cost effective procedure [12]. Other studies have strictly analyzed the costs associated with rotator cuff repair and shoulder arthroscopy. Churchill and Ghorai utilized data from the New York State Ambulatory Surgery Database to investigate cost and operative time differences between mini-open and all-arthroscopic rotator cuff repairs at low, intermediate, and high volume centers. They found that mini-open were significantly less expensive and required significantly less operative time than all-arthroscopic repairs. They also found high volume centers were significantly more expensive than low or intermediate volume centers regardless of repair type [13]. Hearnden and Tennent conducted a cost analysis of arthroscopic subacromial decompression and arthroscopic rotator cuff repair in order to compare costs of these procedures to the national tariff paid in the UK to hospitals for shoulder arthroscopy. They found that a single reimbursement payment for all arthroscopic procedures of *£*1780 was not an accurate payment as subacromial decompression cost *£*1307 and rotator cuff repairs cost *£*2672 [14].

Recently, studies have focused on cost-effectiveness, comparing different techniques of repairing the rotator cuff. Adla et al. used a cost-minimization technique to analyze costs associated with mini-open and all-arthroscopic rotator cuff repair using the direct costs of the two procedures in their hospital. The cost data incorporated consumable goods, cost of operating room time, and the salaries of operating room personnel. They found that both procedures yielded equivalent results with lower costs in the mini-open group making mini-open repair more cost-effective [15]. A decision-analytic model was recently used to evaluate the cost-effectiveness of single-row versus double-row arthroscopic rotator cuff repairs [16]. In this model, the authors developed a decision model with a hypothetical cohort of patients and trace them through various states of health including retear or not, improved or not, and revision surgery or not. The cost data for the analysis of both arthroscopic repair and nonoperative care was derived from previous cost analysis studies [12, 17]. Finally the probabilities of a patient entering each state of health was calculated from previous published data on success and retear rates following single- and double-row repairs [18–20]. The authors found that double-row rotator cuff repair is not cost-effective for any size rotator cuff tears.

Unfortunately, there is an even greater paucity of literature involving cost evaluation of shoulder arthroplasty surgery. The first study was a retrospective cohort study investigating the relationship between clinical outcome and cost of shoulder arthroplasty and surgeon experience. The cost data from this study was derived from the Maryland Health Services Cost Review Commission hospital discharge database. The cost data included the inpatient hospital charges and was descriptively stratified by surgeon experience. This study found that surgeons with higher annual caseloads of total shoulder arthroplasties and hemiarthroplasties have decreased complication rates and hospital length of stays compared with surgeons with lower annual volume [21]. There have been only two cost-effectiveness evaluations in the shoulder arthroplasty literature. Mather et al. analyzed the cost effectiveness of total shoulder arthroplasty versus hemiarthroplasty for the diagnosis of glenohumeral osteoarthritis using a Markov decision model for a cost-utility analysis from a societal perspective [22]. In this model, a sample cohort of patients age 64 was chosen derived from a systematic review [23]. The patients could then enter one of three states (well, revision TSA, or death) following the index procedure. The probability of entering one of these states was determined from the literature. A utility was then calculated from the general health outcome measure, Short Form-36 (SF-36), for each health state and was expressed in quality-adjusted life years (QALY). A utility is a measure of health-related quality of life where 1 represents perfect health and 0 represents death. The cost data for total shoulder arthroplasty, hemiarthroplasty, and revision arthroplasty were estimated using national average Medicare reimbursement for the sum of the Diagnosis-Related Group (DRG) and Current Procedural Terminology (CPT). The outcomes of the analysis were expressed in incremental cost-effectiveness ratio (ICER). ICER: (Cost total shoulder arthroplasty-cost hemiarthroplasty)/(QALY total shoulder arthroplasty − QALY hemiarthroplasty). Using this methodology, the authors concluded that total shoulder arthroplasty is a cost effective procedure, resulting in greater utility for the patient and lower cost to the payer [22]. Coe et al. utilized a similar methodology with a Markov decision model and cost-utility analysis to investigate hemiarthroplasty versus reverse shoulder arthroplasty for the diagnosis of rotator cuff tear arthropathy [24]. The authors found that cost-effectiveness of reverse shoulder arthroplasty depends heavily on the utility gained from the procedure, the utility lost from complications, and the cost of the implant, and that it could be a cost effective operation with an ICER of <$100,000.

 There are several issues associated with the use of Markov decision models and cost-utility analyses in economic evaluations. The inherent limitation in these studies is the requirement of a large number of assumptions to generate the model. All of the assumptions required for a decision model are limited by the quality of the studies used for data. Lower quality studies would obviously limit the conclusions ultimately drawn by the analysis. The results of each model are dependent on the weighting of each variable. These coefficients are determined in reliable models by multiple prospective, randomized controlled trials and in lower quality CEA by assumption and lower quality data. It is critically important to evaluate the data being used to construct the model as each model is only as good as the clinical data it rests upon. The outcomes used for determination of utilities is likely to be obtained from experienced shoulder surgeons whose results may not be applicable to all surgeons who perform shoulder arthroplasty surgery. The cost data obtained from these analyses is based on Medicare reimbursement rates for DRG and CPT codes, which may not reflect to true cost of the operation. While decision models and cost-utility analyses certainly have a role in comparing cost effectiveness of two treatments for a given clinical problem from a societal perspective, they may not be the ideal method for evaluating the true cost of an intervention. The data from current studies in the shoulder surgery literature allow us to draw two simple conclusions: first higher volume centers have lower costs than lower volume centers [21] and the second is that shoulder surgeries are cost effective [12, 15, 16, 22, 23]. While this is useful information, it does not provide a method for determining true costs of shoulder arthroplasty and therefore does not provide surgeons with a methods for determining and reducing costs while maintaining the highest quality of care.

 As the bundled payment initiative approaches as part of the Patient Protection and Affordable Care Act, shoulder surgeons have the opportunity to develop an improved method of determining the true cost of shoulder arthroplasty to become active leaders in accountable care organizations [1]. Without knowledge of the true cost of caring for a shoulder arthroplasty patient, it is impossible for CMS to determine a value for the bundled payments.

## 4. Conclusion

 In order to become leaders in decreasing cost in shoulder arthroplasty surgery, surgeons need to have a methodology for collecting outcomes and cost data. The current literature does not provide a methodology for surgeons to determine the cost of shoulder arthroplasty. Kaplan and Porter advocate the use of “time-driven activity-based costing” which requires that providers estimate two parameters at each step in the health care process: the cost of each of the resources used and the quantity of time spent with each resource [25]. This methodology may provide an additional method to determine the total two-year cost of total shoulder arthroplasty.

 Shoulder surgeons are positioned to become the director of the bundled payment as surgeons ultimately initiate the episode of care through their assessment to offer a patient surgery based on his/her own abilities and expertise. Furthermore, surgeons efforts to measure outcomes and follow patients postoperatively put them in the unique position to know the drivers of successful outcomes following the intervention. With the additional knowledge of the costs associated with the entire episode of care, this naturally places the surgeon in the position of directly allocating resources while maintaining or improving on the outcomes obtained.

One could argue since the surgeon fee is only a fraction of the cost of surgery, a hospital and its administration in the role as gatekeeper of the bundled payment could bid out physician services in a similar manner to implants and equipment.

 There are many methods to analyze cost in shoulder surgery and all of them have a role in gaining useful information regarding the costs of delivering care. The bundled payment initiative provides an opportunity for surgeons to take an active role in cost containment. The ultimate goal for surgeons it to decrease cost while maintaining or improving the quality of care delivered. We hypothesize that simply the awareness of costs associated with the episode of care of shoulder arthroplasty surgery will naturally cause surgeons to increase efficiency of care and decrease associated costs. It is critical that research continues to be performed regarding costs in shoulder arthroplasty to provide every shoulder surgeon with a method to collect cost and outcomes data.
